# Smart and Functional Conducting Polymers: Application to Electrorheological Fluids

**DOI:** 10.3390/molecules23112854

**Published:** 2018-11-02

**Authors:** Qi Lu, Wen Jiao Han, Hyoung Jin Choi

**Affiliations:** Department of Polymer Science and Engineering, Inha University, Incheon 22212, Korea; 22172314@inha.edu (Q.L.); 22151728@inha.edu (W.J.H.)

**Keywords:** conducting polymer, composite, electrorheological, smart fluid, viscoelastic

## Abstract

Electro-responsive smart electrorheological (ER) fluids consist of electrically polarizing organic or inorganic particles and insulating oils in general. In this study, we focus on various conducting polymers of polyaniline and its derivatives and copolymers, along with polypyrrole and poly(ionic liquid), which are adopted as smart and functional materials in ER fluids. Their ER characteristics, including viscoelastic behaviors of shear stress, yield stress, and dynamic moduli, and dielectric properties are expounded and appraised using polarizability measurement, flow curve testing, inductance-capacitance-resistance meter testing, and several rheological equations of state. Furthermore, their potential industrial applications are also covered.

## 1. Introduction

**Figure 1:**
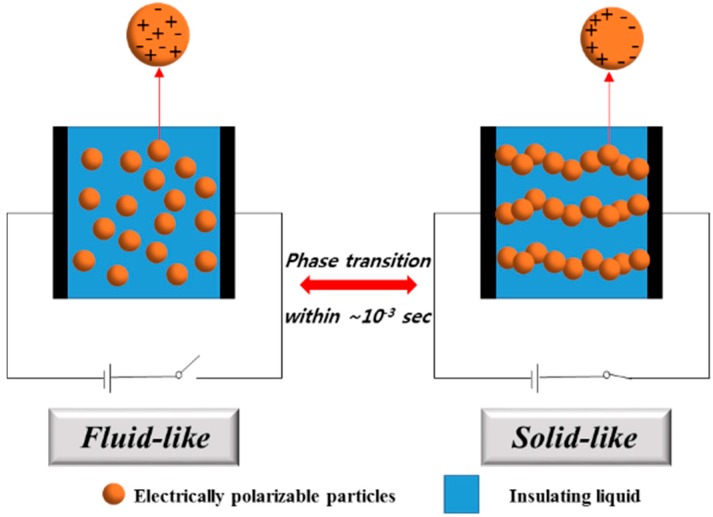
Schematic diagram of electrorheological (ER) behavior with an external electrical field.

Smart functional materials that can detect and identify external (or internal) stimuli, including electricity, light, heat, magnetic fields, stress, strain, and chemicals [1], have gained attention due to their various engineering applications, such as vibration controls [2], detection [3], electronics [4], and drug delivery [5]. Among these smart functional materials, electrorheological (ER) suspensions consisting of electro-responsive particles and an insulating fluid, such as silicone or mineral oil [6,7,8], play a crucial role. By engaging an electric field, the electro-responsive functional particles begin to polarize within milliseconds and the initially dispersed particles aggregate to build a chain form, following an external electrical field direction because of an induced dipolar attractive force. Thus, the microstructure of the ER suspension changes from a liquid-like to a solid-like form, as shown in Figure 1. Therefore, their physical and rheological behaviors, such as steady shear viscosity, shear stress, dynamic modulus, and yield stresses, and even stress relaxation changes according to the applied electric fields; however, these changes can be reversed by removing the field. Most ER fluids suit the Bingham fluid model, which illustrates that a fluid has its own yield stress, and the flow motion of the fluid impedes when an input external shear stress is lower than the yield stress [9]. ER fluids with this controllable and tunable phase change have a very good market prospect and wide applications, such as dampers [10], ER haptic devices [11], shock absorbers, microfluidics [12,13,14], clutches [15], ER finishing devices [16], and tactile displays [17].

ER fluids have been the subject of research and exploration since their discovery [18]. Furthermore, research to find fibrillation structures between two applied electrodes with an apparent increase of the viscosity of ER fluids have also been conducted. Klass and Martinek [19] introduced a dielectric principle, and a water-associated electrical double-layer model, to explain that the critical factor of the ER effect was the molecule in the wet-base ER fluids. The particles were polarized and distorted when the extra electric field was applied and then the water molecules made an attractive bridge between dispersed particles, thereby showing a higher surface tension.

Furthermore, ER materials cover a wide range of organics and inorganics particles, including polymers and their organic or inorganic composites, with their particle size ranging from nanometers to micrometers with multiple shapes, such as spherical, fibrous, sheet, and rod-like [20,21].

In this study, we focus on anhydrous ER materials with various conducting polymers and their nanocomposites. Conducting polymers possess conjugated repeating units, which can be appraised by some important variables, such as ionization potential, electron affinity, band gap, and bandwidth [22,23,24]. By regulating these parameters the thermal, electrical, and optical properties of polymers can be easily enhanced [25,26]. In particular, the electrical conductivity and thermal or mechanical stability of nanocomposites, which have improved mechanical property, dispersion stability, or physical characteristics, and can be easily controlled by various fabrication techniques [27].

Sundry conducting polymers have been employed as ER materials, including polyaniline (PANI) [28,29], polypyrrole (PPy) [30], poly(*p*-phenylene) (PPP) [31,32,33], poly(3,4-ethylenedioxythiophene) (PEDOT) [34], and so on. This study focuses on the ER effect of conducting polymeric materials and their composites, including synthesis methods and typical characteristics, using various rheological measurement methods. Furthermore, according to the viscoelastic behaviors, such as flow curves, shear viscosity, yield stresses, and dynamic modulus, the ER effect of polymeric materials dispersion is compared under an external electrical field with various strengths.

## 2. Polymeric ER Materials

Oxides or non-oxide inorganic particles were introduced in ER fluids at the initial stages of study. However, the dispersion stability was poor because of the large density variation between the dispersed particles and the base oil. Therefore, organic particles gained attention, especially conducting polymers, which are provided with conjugated π bonds along with a high dielectric constant, thereby exhibiting excellent polarization capabilities under applied electric field strengths. Moreover, these types of polymers can be comprehensive as ER materials because they have highly polarizable functional groups on the molecular backbone.

### 2.1. PPy

Among the various conductive polymers, PPy is a research hotspot because of its high electrical conductivity, simple method of preparation [35], ease of surface modification [36], excellent environmental stability [37], and ion-exchange capacity [38]. In addition, it can be easily produced in a large quantity at a room temperature using various solvents, including water. PPy is an excellent conductive polymer; thus, it can be applied in ER fluids.

**Figure 2:**
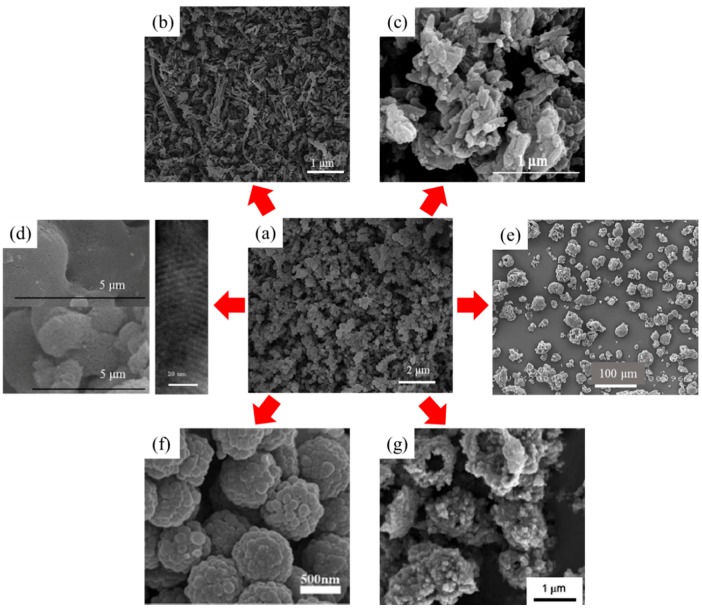
SEM morphologies of polypyrrole (PPy) and its composites: (**a**) granular PPy and (**b**) PPy nanofibers, reprinted with permission from [39]. Copyright Elsevier, 2011; (**c**) PPy-HNT particles, reprinted with permission from [41]. Copyright Springer Nature, 2014; (**d**) PPy/MCM-41 particles, reprinted with permission from [43]. Copyright Elsevier, 2010; (**e**) PPy-polycaprolactone (PPy-PCL) composites, reprinted with permission from [44]. Copyright Springer Nature, 2007; and, core-shell structures of (**f**) PPy-silica, reprinted with permission from [48]. Copyright RSC Publishing, 2016 and (**g**) PPy-TiO_2_, reprinted with permission from [49]. Copyright Springer Nature, 2011.

Kim and Song [33] dispersed PPy in mineral oil as an ER fluid and controlled the amount of oxidant and surfactant while synthesizing PPy particles to investigate the impacts on their ER effect. They observed that as the amount of oxidant and surfactant increases the yield stress increases at the initial stage but decreases after passing culmination because of the enhanced particle polarization. Using thermo-oxidative treatment in air, Xia et al. [39] tuned the electrical conductivity of the PPy nanofibers (shown in Figure 2b) to that of a nanofiber-based ER fluid. Its yield stress and shear modulus were stronger compared to those from a typical granular-shaped PPy based ER suspension, because the fiber-like particles can ameliorate the dispersion stability of the suspension. Many studies have been conducted on preparing conducting PPy-based composite materials to surmount the poor processability of PPy particles.

Moreover, while the morphology of the conducting polymers is well-known to be related to their synthetic conditions, such as temperature, pH, oxidant species, oxidant/monomer ratio, polymerization type, and so on, shape- or size-related morphology of the dispersed particles became an important parameter in the rheological output of ER fluids. Specifically, regarding the effect of a hierarchical form on ER properties, sea urchin-like conducting polymeric particles are known to exhibit both better sedimentation stability than those with a smooth surface and better rheological behaviors. They are expected to show larger drag forces and inter-particular frictions with their increased surface area compared to that of the smooth-surfaced particles. Therefore, an improved ER efficiency with larger yield stress can be expected in the same experimental conditions. Moreover, compared to the ER fluid of spherically shaped particles, the systems with fiber-like particles were also observed to exhibit better ER characteristics and enhanced suspension stability because the elongated shape possesses a bigger induced-dipole moment and greater inter-particle interaction.

Concurrently, clays are widely used to compose polymer composites not only because of their large specific surface area and good mechanical properties, but also because of their chemical, thermal, and dimensional stability and low cost [40]. Halloysite (HNT) is a type of clay mined from natural deposits and has a predominantly hollow tubular structure. HNT has been widely used in electronics, catalysis, separation, and functional materials because of its uniquely versatile surface features. Jang et al. [41] synthesized PPy/HNT nanocomposites (Figure 2c) and observed that adding HNT is an effective method to improve PPy conductivity and properties of PPy-based ER fluids. Furthermore, montmorillonite (MMT) clay is widely used in conducting polymer nanocomposites because their particle size is smaller than 10 μm and they can be easily intercalated. Cations, such as Na^+^ and Ca^2+^, in its lamellar surface enable interactions with monomers ensuring reasonable conductivity of the associated electric signal. Following testing, Kim et al. [42] observed that PPy-dodecylbenzenesulfonic acid (DBSA)/clay nanocomposites have better insulation and thermal stability. PPy was also inserted into hexagonal channels of pore-expanded MCM-41 [43], whose insulating channel can improve the ER response of the PPy suspension (Figure 2d).

Not only clay, but also conventional polymers were introduced in conducting polymer/clay nanocomposites to overcome the limitation of poor processability, prepare conducting polymer-based composite materials, and improve the sedimentation of ER suspensions. Kim et al. [44] assembled PPy-polycaprolactone (PPy-PCL) composites (Figure 2e) to improve the mechanical and electrical properties of PPy, along with enhancing their ER response. Furthermore, Kim and Park [45] reported an ER response by coating PPy on polyethylene particles, thereby enhancing polarization by increasing the particle surface conductivity.

Moreover, compared to single-component counterparts, core-shell structured particles often exhibit better physical and chemical properties. A conducting shell can be polarized instantaneously; thus, its ER response can be improved. In addition, an insulating core or an insulating shell in the composite particles is designed to obtain a lower current density, by which the practicability and environmental protection of the materials can be improved [46,47]. Kim et al. [48] synthesized PPy-coated silica core-shell structured nanoparticles (Figure 2f), while Sedlačík et al. [49] synthesized titanium oxide (TiO_2_)/PPy core-shell structured nanoparticles (Figure 2g) and analyzed the steady-state and oscillatory flow behaviors. Both sets of results showed that the novel core-shell structure possessed predominant maneuverability and ER response.

### 2.2. PANI

**Figure 3:**
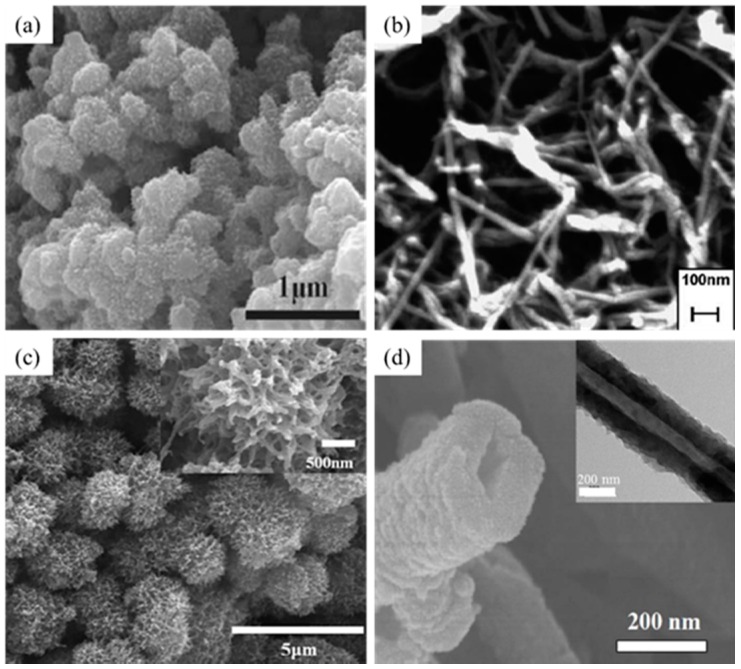
SEM images of synthesized polyaniline (PANI): (**a**) spheres, reprinted with permission from [55]; Copyright Spring Nature, 2013; (**b**) fibers, reprinted with permission from [52]. Copyright American Chemical Society, 2009; (**c**) urchin-like, reprinted with permission from [56]. Copyright John Wiley and Sons, 2009; and (**d**) nanotubes, reprinted with permission from [57]. Copyright American Chemical Society, 2006.

Among various whole conductive polymers, PANI is considered an essential candidate material for ER fluids because of its ease of synthesis, low price, chemical and thermal stabilities, and sensitive response to easily controllable electrical conductivity, which can be steered by a reversible doping/dedoping process [50]. Most morphologies of a synthesized PANI are irregular. However, using some methods, such as interfacial polymerization [51] and rapid mixing method [52], and using a surfactant or template [53] to direct PANI growth, we can obtain various shapes of fibers, tubes, spheres, and even urchin-like particles (shown in Figure 3).

**Scheme 1:**
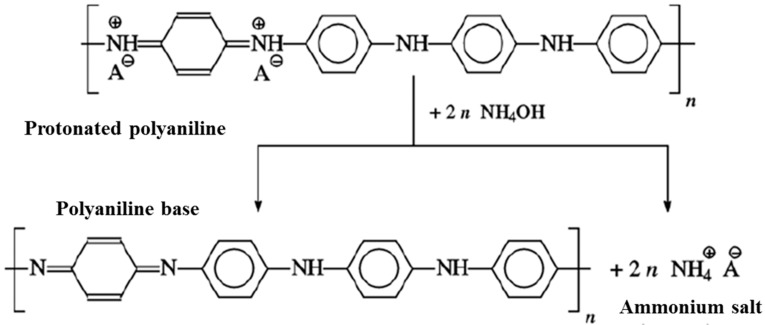
Conductive protonated PANI is converted to a non-conducting PANI base by deprotonation with ammonium hydroxide.

For ER fluid applications, the high conductivity of PANI is undesirable because of the high leakage currents passing through the suspension with the electric field [54]. Thus, the protonated PANI needs to be changed to a PANI base by treating it with an ammonium hydroxide solution. The electrical properties of the protonated PANI and its corresponding bases can be controlled at a molecular level by partial protonation and acid selection of substitution of the PANI ring, or copolymerization of aniline with substituted aniline or other monomers, as shown in Scheme 1. In addition to the powder, PANI can be deposited on almost any object in the reaction mixture during aniline oxidation, or as a colloid when the reaction mixture contains a stabilizer.

**Figure 4:**
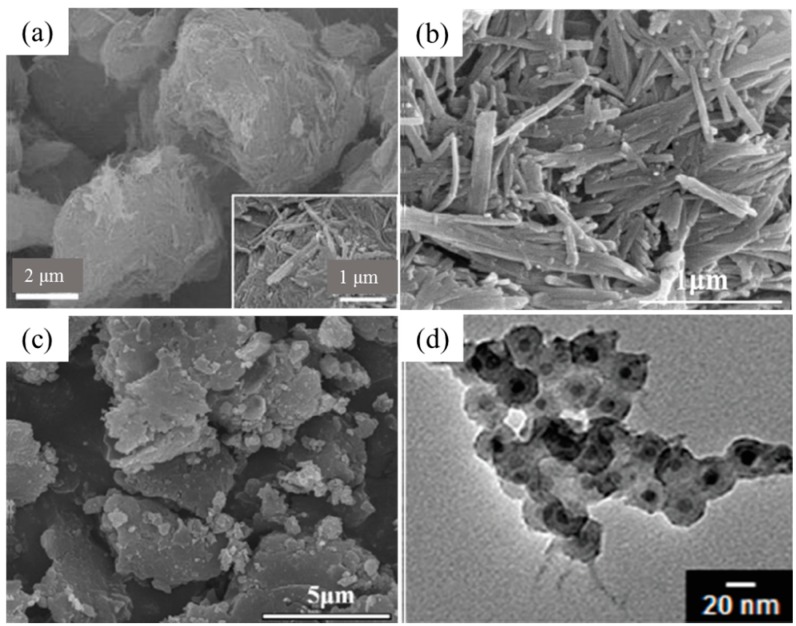
SEM and TEM photos of: (**a**) PANI/ATP, reprinted with permission from [59]. Copyright Elsevier, 2017; (**b**) PANI/SPL, reprinted with permission from [61]. Copyright Elsevier, 2015; (**c**) PANI/laponite, reprinted with permission from [63]. Copyright Elsevier, 2015; and (**d**) Fe_3_O_4_/SiO_2_/PANI nanoparticles, reprinted with permission from [64]. Copyright Royal Society of Chemistry, 2017.

**Figure 5:**
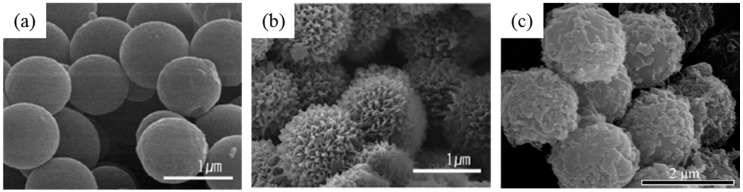
SEM images of: (**a**) smooth PANI@PS and (**b**) urchin-like PANI@PS, reprinted with permission from [65]. Copyright RSC Publishing, 2015; and (**c**) PGMA/PANI composite, reprinted with permission from [67]. Copyright Elsevier, 2013.

To improve the ER response of PANI particles and obtain an appropriate semiconducting regime for an ER application, multifarious inorganics, such as clays, TiO_2_, silica, and BaTiO_3_, and even magnetic materials, such as carbonyl iron or Fe_3_O_4_ (shown in Figure 4) were introduced. Furthermore, some non-conducting core polymers form a core-shell structure with PANI as a shell (shown in Figure 5). For example, as one type of clay, attapulgite (ATP) or palygorskite, which is a kind of crystalline hydrated magnesium silicate with a fiber shape, has been introduced for PANI/clay composites. It not only possesses a large specific surface area but also cation exchange capacity and a reactive hydroxyl group on its surface [58]. Han et al. [59] synthesized PANI/ATP composite materials and then estimated their ER responses, observing that they were a feasible and efficacious method for obtaining good mechanical strength, and easy processing of composite ER material. Furthermore, sepiolite (SPL), another species of natural silicate clay with a fibrous morphology that has a large surface area and porosity showing its mechanical reinforcing capability [60], has also been introduced. Owing to that, Jang and Choi [61] used SPL synthetizing with PANI to obtain a neoteric ER material. As a multi-functional physical cross-linker, laponite, a synthetic clay material, which was adopted to stabilize emulsions because of its uniform and small dimensions, can lead to non-covalent cross-linking on the clay/polymer interface [62]. Jun et al. [63] combined PANI and laponite confirming that this composite displayed a typical ER behavior. Noh et al. [64] reported neoteric Fe_3_O_4_/SiO_2_/PANI nanoparticles, which can be applied in both ER and magnetorheological (MR) fluids. In addition, they observed that the ER and MR responses were significantly improved under both electric and magnetic fields.

Moreover, PANI has also been applied comprehensively with various polymeric shells. It is not sufficiently sophisticated to form a PS/PANI nanosphere, due to π-π stacking attractive interactions between an aniline monomer and sulfonating polystyrene (PS) using concentrated sulfuric acid. Kim et al. [65] compared two types of PS/PANI-based ER fluids whose PANI shell was either smooth or urchin-like. In Lee et al.’s work [66], the glycidyl methacrylate (GMA) was used to graft an aniline monomer with poly(methyl methacrylate) PMMA to form a PANI/PMMA core-shell structure. However, in Zhang et al.’s report [67], the core-shell PGMA/PANI composites were synthesized and applied in ER fluids.

### 2.3. Other Conducting Polymers

**Figure 6:**
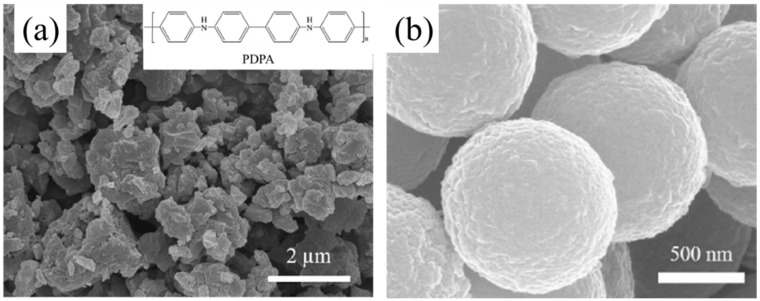
SEM images of (**a**) Poly(diphenylamine) (PDPA), reprinted with permission from [70]. Copyright Elsevier, 2017 and (**b**) PDPA/PS core/shell structure, reprinted with permission from [71]. Copyright Elsevier, 2017.

In addition to either PPy or PANI, various other conducting polymers and their derivatives were applied in ER fluids. Although PANI has its own advantages in terms of controllable conductivity, environmental stability, and simple synthesis, it also exhibits limitations, such as poor solubility in various organic solvents. To overcome these defects, various derivatives were introduced [68]. Poly(diphenylamine) (PDPA) as a derivative of PANI, possesses not only a relatively low conductivity, which helps avoid cause short-circuiting and can be directly applied in an ER fluid, but also produces improved solubility in organic solvents with better thermal stability [69]. Kim et al. [70,71] at first synthesized normal PDPA nanoparticles for an ER fluid, and then further fabricated a novel core-shell-type PDPA/PS structure (shown in Figure 6), thereby observing that both these materials were suitable for ER fluids.

**Scheme 2:**
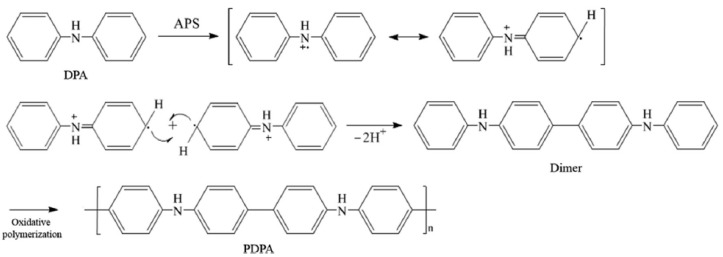
Schematic diagram of PDPA synthesis via an oxidative polymerization.

The chemical formula of a *p*-phenylenediamine (PDA) is similar that of an aniline. It inspires its ER application with its polymeric form [72] along with its versatile synthetic methods to prepare poly(*p*-phenylenediamine) (PPDA, whose schematic diagram is shown in Scheme 2) particles as compared to PANI. Plachy et al. [73] tried to modify PPDA by heat treatment to achieve appropriate conductivity; however, they observed that the ER efficiency decreased as the carbonization temperature increased. Cao et al. [74] fabricated poly(*p*-phenylenediamine)/graphene oxide (PPDA/GO) composite particles, and observed that both PPDA/GO particles and PPDA-based ER fluids exhibited significant ER characteristics.

It can be also noted that by introducing proper substituents on polymeric backbones, functionalized PANI could be fabricated, providing extra tuning capability of their chemical and electrical characteristics [75,76].

**Figure 7:**
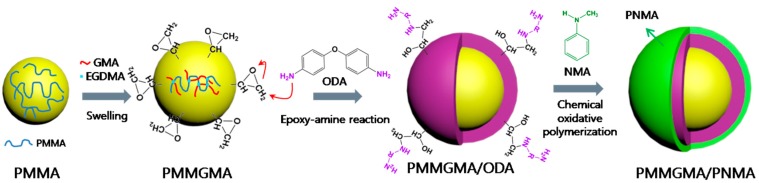
Schematic diagram of fabrication of poly(methyl methacrylate)/poly(*N*-methylaniline) (PMMA–PNMA) core-shell particles, reprinted with permission from [77]. Copyright Elsevier, 2015.

Recently, poly(*N*-methylaniline), as a derivative of PANI, has piqued the interest of many researchers as, compared to PANI, it possesses much lower electrical conductivity. Thereby, it could be directly applied to the ER fluid without further treatment. Moon et al. [77] used a simple method to fabricate a core-shell typed poly(methyl methacrylate)/poly(*N*-methylaniline) (PMMA-PNMA). Figure 7 shows the synthetic fabrication of the PMMA-PNMA particles. Initially, the surface of the PMMA microspheres, which were fabricated through a dispersion polymerization process, was treated with glycidyl methacrylate, ethylene glycol dimethacrylate, and oxydianiline as swelling, crosslinking, and chemical-grafting agents, respectively. Further, the PNMA shell was coated by chemical oxidation polymerization. Their ER suspension based on the PMMA-PNMA particles demonstrated conventional ER behaviors in both simple shear flow and dynamic oscillation measurements for several different electric field strengths applied.

Polyindole (PIN) has attracted huge attention due to its good thermal stability and high redox activity [78]. Indole is composed of a benzene ring connected to the heterocyclic pyrolytic ring; thus, it was anticipated that it would show the properties of both PPy and PPP. Park et al. [79] synthesized emulsion-polymerized PIN nanoparticles and used these as an ER material. Sari et al. [80] synthesized PIN/polyethylene composites and observed that they were thermally more stable than PIN, and the ER suspensions was observed to be increasing with increases in the electric field.

**Figure 8:**
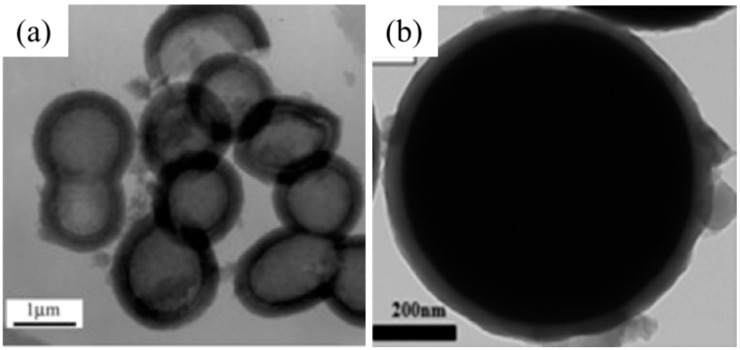
TEM images of (**a**) hollow poly(*O*-anisidine) (POA), reprinted with permission from [81]. Copyright IOP Publishing, 2011 and (**b**) POA/silica core/shell structure, reprinted with permission from [82]. Copyright IOP Publishing, 2017.

Poly(*O*-anisidine) (POA), with a methoxy functional group (−OCH_3_) at an ortho position, lowers the mobility of electric charge carriers following the polymeric backbone, thereby lowering the electrical conductivity. Recently, POA was adopted as an ER material. Cheng et al. [81] used chemical oxidation polymerization to synthesize hollow POA microspheres with 1 μm diameter in a sphere-like form. The hollow POA particles had low density, which is an advantage for a high-stability dispersed phase ER fluid. Furthermore, Lee et al. [82] fabricated a core-shell structure of POA-silica particles using *N*-[(3-trimethoxylsilyl)-propyl] aniline (PTMSPA) grafting agent and defined their ER properties (as shown in Figure 8).

Polyacene quinone radicals (PAQRs) are also a type of semi-conductive polymers, which possess the p-conjugated structure. They have been widely used and studied because of their better thermal stability, environmental stability, and the characteristic that their conductivity can be controlled by polymerization conditions (such as temperature, acenes, and anhydrides). Wang et al. [83] synthesized hollow PAQR sub-microspheres by a modified solvo-thermal method and then observed that their ER fluid exhibited conventional ER properties. Poly(naphthalene quinone) (PNQR) has a similar chemical p-conjugated structure as PAQR, and so also has an electrical conductivity and can be applied as an ER fluid material. Cho et al. [84] synthesized PNQR from the Friedel–Craft acylation and its flow behaviors when suspended in the silicone oil were analyzed using rheological equations of state from Bingham, De Kee-Turcotte, and Cho-Choi-Jhon (CCJ) equations.

Meanwhile poly(3,4-ethylenedioxythiophene)(PEDOT):poly-(styrenesulfonic acid) as a derivative of polythiophene, has attracted considerable attention because of its several advantages as a conducting polymer, such as processability in aqueous solutions, and better thermal and structural stability [85]. An et al. [86] fabricated core/shell typed polystyrene (PS)/PEDOT microspheres and reported their ER behaviors under an electrical field. On the other hand, Erol et al. [87] synthesized core/shell typed nano-rods with a titania core and conducting polymeric PEDOT shell. They were fabricated via covalent bonding to obtain a thin polymeric shell, making the interfacial interactions between the two components stronger. They found that the nanorod-shaped TiO_2_/PEDOT particles exhibited improved ER performance and storage moduli, as well as larger creep-recovery after stress loading, when compared to their particulate morphology.

Furthermore, to solve problems such as high conductivity and low thermal stability of PANI, aniline is often copolymerized with other conductive polymer monomers to form a more functional conductive polymer. Cho et al. [88] synthesized poly(aniline-*co*-pyrrole) (PAPP) particles (aniline:pyrrole = 1:1) via a chemical oxidation polymerization technique. It was proved that the copolymer has better thermal stability and more suitable conductivity than PANI as the dispersed phase in ER fluid. Pandey et al. [89] fabricated poly(aniline-*co*-*O*-anisidine) with a lower value of electrical conductivity by explaining the incorporated *O*-anisidine moieties in PANI backbones. Trlica et al. [90] also reported a poly(aniline-*co*-1,4-phenylenediamine) particles-based ER suspension with an applied external electric field, along with electrical characteristics of an ER suspension.

### 2.4. Composites

Various inorganic-conducting polymers have been introduced for their ER applications, and among these, recently graphene has attracted great interest in the fields of capacitors, electronic devices, sensors, and composites due to its unique structure and electrical characteristics, even in ER fluids [91]. Although graphene oxide (GO) which is the oxidized form of graphene has many defects during its preparation process, it contains functional components such as an hydroxyl group, epoxy group, and carboxyl group, making it more easily dispersed in water [92]. By simply compounding with other polymers, it can be modified to have multiple functions. Zhang et al. [93] synthesized PANI/GO composites by an in-situ oxidation polymerization method with a stable GO and reported its composite particle-based ER suspension showed a general ER property. On the other hand, Min et al. [94] fabricated core-shell typed PMMA/GO microspheres via a Pickering emulsion polymerization method with the GO as the solid surfactant, and studied their ER response. Cao et al. [74] synthesized poly(*p*-phenylenediamine)(PPDA)/GO composite particles via an in-situ oxidation polymerization technique of *p*-phenylenediamine in GO suspension, observing improved typical ER behavior when compared to PPDA particle-based ER fluid.

Furthermore, carbon nanotubes as another candidate for ER fluids have received a lot of consideration [95,96,97], although compared with single-walled carbon nanotubes (SWNT), multi-walled carbon nanotubes (MWNT) have been more widely used in the ER field due to their lower electrical conductivity. Jin et al. [98] produced MWNT-adsorbed PS and PMMA particles using different surfactants, such as anionic sodium dodecyl sulfate and sodium dodecylbenzene sulfonate, and cationic cetyl trimethylammonium bromide. Not only conductive carbon materials, but also many metal oxides, can be compounded with conductive polymers to form multifunctional materials showing remarkable ER effects. Fang et al. [99] synthesized magnetic nanoparticles (Fe_3_O_4_) together with a conductive PPy using a simple fabrication method, and applied it to both ER and MR fluids.

### 2.5. Poly(Ionic Liquid)s

**Figure 9:**
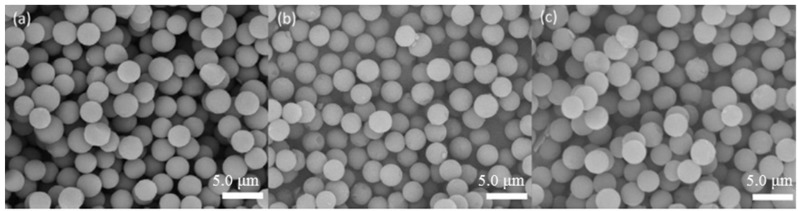
SEM photos of poly(ionic liquid)s (PIL) particles: (**a**) poly((*p*-vinylbenzyl trimethylammonium) (VBTMA)^+^CF_3_SO_3_); (**b**) poly(VBTMA^+^ (CF_3_SO_2_)_2_N^−^); and (**c**) poly((*p*-vinylbenzyltriethylammonium bis(trifluoromethane sulfonimide) (VBTEA)^+^ (CF_3_SO_2_)_2_N^−^), reprinted with permission from [102]. Copyright Elsevier, 2016.

Poly(ionic liquid)s (PILs), initially evaluated as ion conductive polymers, refer to a new type of polymers in which ionic liquids (ILs) are linked through polymeric backbones to form macromolecular structures. They have attracted huge scientific interest not only because they have the suitable electrical conductivity and thermal stability from the ILs, but also because, like polymers, they have the advantages of good solid morphology, simple processing and good mechanical properties [100,101]. To activate the ER effect, classical polyelectrolytes need to adsorb a tiny amount of water to boost the local ion movement which will cause chemical corrosion, dielectric breakdown, and limited operation at higher temperatures. Nevertheless, because of fluoric counter-ions including tetrafluoroborate (BF_4_^−^), hexafluorophosphate (PF_6_^−^), and fluorinated imide ((CnF_2*n*+1_SO_2_)_2_N^−^), PILs are generally organophilic and insoluble in water. Using a microwave-assisted dispersion polymerization technique, Dong et al. [102] synthesized mono-dispersed PIL particles possessing a styrenic backbone and different sizes of cationic/anionic parts (shown in Figure 9) and reported their enhanced anhydrous ER characteristics. Then they further synthesized several mono-dispersed poly[*p*-vinylbenzyl trimethylammonium] PIL microspheres possessing a similar diameter but different counter-anions. This showed that PILs with a smaller volume or counter-ion size exhibited higher ER behavior compared to PILs possessing organic counter-ions or inorganic counter-ions alone [103].

## 3. ER Characteristics

### 3.1. Direct Observation

**Figure 10:**
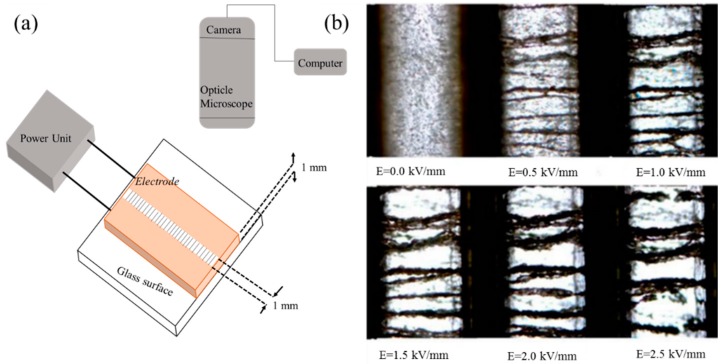
(**a**) Schematic illustration of polarizability measuring device; (**b**) optical microscope (OM) images showing the polarization of nanocube-TiO_2_/poly(3-octhlthiophene)-based ER fluid, reprinted with permission from [104]. Copyright John Wiley and Sons, 2016.

The electrical stimuli response characteristics of ER fluids can be directly detected by an optical microscope (OM) with an external electric field [84]. The polarization measuring device is presented in Figure 10a. The ER suspension was placed in the center of a transparent substrate connected to a DC high voltage power supply, and micro-structural changes were captured by the OM. Sever and Unal [104] prepared an ER fluid by dispersing nanocube-TiO_2_/poly(3-octhlthiophene) in silicone oil and observing its polarizability and potential ER performance with this OM device technique. The particles were suspended randomly similar to a liquid state when E = 0 kV/mm, while they were electrically polarized and migrated to organize a columnar structure parallel to a field direction when E ≠ 0 kV/mm (Figure 10b). The formation of this chain like-columnar structure caused ER fluid to exhibit a solid-like behavior, which was attributed to the polarization of the particles [8]. Furthermore, this columnar structure was found to be stronger and thicker, in accordance with the increment in applied E range from 0.5–2.5 kV/mm and could be maintained as long as an external electrical field applied, indicating that the increased E caused a tighter columnar structure. These results are consistent with OM studies carried out for various ER fluid materials [105,106,107].

### 3.2. Flow Curve Behaviors

Stable steady shear flow curves in ER fluids can be established via a typical rotation rheometer test, like the controlled shear rate (CSR) method, which provides a basis for observing the yield stress of ER suspensions and electrical stimulus response behavior [21,108]. The flow curve of ER fluid produced from this CSR method exhibits shear stress (*τ*) and shear viscosity (*η*) as a function of shear rate $\left(\dot{\gamma }\right)$.

In general, two types of interactions occur between dispersed ER particles in a steady shear flow; the electrostatic interaction caused by an external electrical field and a hydrodynamic interaction induced by a steady shearing flow [109]. The shear flow field results in shear deformation of the chain structures formed under an electric field; however, the broken structure tends to rebuild the chain simultaneously under an applied external electrical field. These phenomena of destruction and reorganization depend on the applied shear stresses and the electric field strengths. At a low shear rate regime, the electro-static attractive interaction becomes prominent, allowing the chain structure to reduce the effect from shear deformation [110]. As a shear rate increases, the breaking rate exceeds the recombination rate, so that the chain structure will eventually be destroyed and will exhibit a liquid-like behavior. However, a stronger electric field can result in a stronger electrostatic interaction which is sufficient to resist shear deformation, allowing the solid-like behavior of the ER fluid to re-emerge.

Based on the electrical stimuli-response behaviors of ER fluids in a shear flow field, many models have been introduced to qualitatively explain the flow curve. The Bingham model (Equation (1)), the simplest equation with two parameters, has been extensively employed as a viscoelastic equation to fit steady shear behaviors exhibited in ER fluids along with various suspensions and MR fluids [111]:

(1) $$\begin{array}{ll} \tau =\tau_{y}+\eta \dot{\gamma } & \tau \geq \tau_{y}\\ \dot{\gamma }=0 & \tau <\tau_{y} \end{array}$$

**Figure 11:**
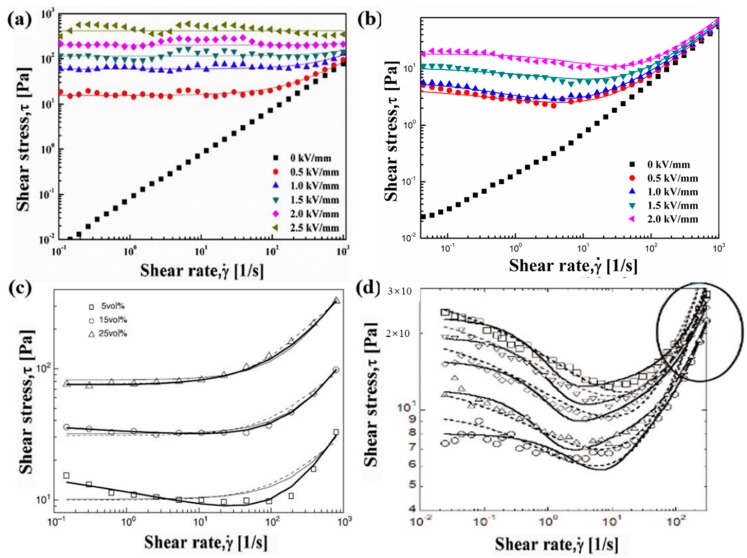
Shear stress vs. shear rate for: (**a**) PS/PANI-based ER suspension (solid lines for Bingham equation), reprinted with permission from [112]. Copyright Elsevier, 2017; (**b**) PANI@ATP-based ER suspension (solid lines for CCJ equation), reprinted with permission from [59]. Copyright Elsevier, 2017; (**c**) poly(naphthalene quinone)-based ER fluid (dashed lines for Bingham equation, solid lines for De Kee-Turcotte equation, and bold solid lines for CCJ equation), reprinted with permission from [84]. Copyright American Chemical Society, 2012; and (**d**) PMMA/PANI-based ER fluid (dotted and solid lines for CCJ equation and four-parameter equation), reprinted with permission from [117]. Copyright Elsevier, 2005.

In Equation (1), $\dot{\gamma }$ is a shear rate; *η* is a shear viscosity; *τ* and $\tau_{y}$ are shear and yield stresses, respectively. Piao et al. [112] explained shear stress behaviors of PS/PANI-based ER suspension using this Bingham fluid model, in which the flow curve, as shown in Figure 11a, was fitted by this model quite well. Its shear stress was observed to increase linearly with increased shear rate when E = 0 kV/mm, which is similar to that of Newtonian fluids. Under an applied electrical field, the ER suspension demonstrated increments in shear stresses and exhibited a plateau behavior because of the formation of the columnar structure.

However, in the case of Figure 11b [59], shear stresses of the PANI@ATP-based ER suspension presented a weak concave shape in a low shear rate area, passed through a minimum value, and once again increased. This behavior could be explained by the fact that the reconstituted structure is not as complete as the structure formed before the steady shearing flow applied. Thus, they adopted the Cho-Choi-Jhon (CCJ) equation [84], which is an empirical formula with six parameters to express the ER characteristics in a whole shear rate range. This CCJ equation is given as follows:

(2) $$\tau =\frac{\tau_{y}}{1+{\left(t_{1}\dot{\gamma }\right)}^{\alpha }}+\eta_{\infty }\left(1+\frac{1}{{\left(t_{2}\dot{\gamma }\right)}^{\beta }}\right)\dot{\gamma }$$

The first term corresponds to the decrease in the shear stress at the low shear rate area, and the next term depicts a shear-thinning property at the high shear rate area. The *t*_1_ and *t*_2_ parameters are time constant, and $\eta_{\infty }$ is a shear viscosity at the infinite shear rate. The exponent *α* represents the shear stress decrease, and *b* is located between 0 and 1. This model was observed to be a good indication of the overall behavior of various ER fluids [61,87,113], including the reduced shear stress phenomenon.

In addition, the De Kee-Turcotte equation (Equation (3)) and Herschel-Bulkley equation (Equation (4)), as a modified version of the Bingham equation, have also been applied for various ER suspensions [114,115]:

(3) $$\tau =\tau_{0}+\eta_{1}\dot{\gamma }e^{-t_{1}\dot{\gamma }}$$

(4) $$\tau =\tau_{0}+m{\dot{\gamma }}^{n}$$

where *t*_1_ is a time constant, m is a consistency index, and n is a flow index. Cho et al. [84] constructed the flow curve of a poly(naphthalene quinone)-based ER suspension for different particle concentration and electric field strengths, and interpreted its steady-state flow properties using three different equations from the Bingham, De Kee-Turcotte, and CCJ models. As presented in Figure 11c, the CCJ equation fitted the decrement of shear stress better for a shear rate range from 0.01–100 s^−1^; however, significant deviation was found at a low shear rate region compared to other two equations, especially for a lower volume fraction of poly(naphthalene quinone)-based ER fluid.

Based on the above models, Seo and colleagues [116] proposed a new rheological model (Equation (5)) that can depict the flow behavior of the ER suspensions over the full range of shear rates with four parameters.

(5) $$\tau =\tau_{sy}\left(1-\frac{\left(1-\exp\left(-a\dot{\gamma }\right)\right)}{\left(1+{\left(a\dot{\gamma }\right)}^{\alpha }\right)}\right)+\eta \dot{\gamma }$$

This four-parameter model was introduced to predict static yield stress, while the CCJ and Bingham models were based on dynamic yield stress. In Equation (5), *a* is a time constant, $\tau_{sy}$ represents a static yield stress, and α is a power-law index relating to the shear thinning. In Figure 11d, Seo and Seo [117] analyzed the flow curves of PMMA/PANI-based ER suspension using the CCJ equation and their proposed equation. At a low shear rate regime, the shear stresses predicted by the new model converged to yield stress, while the prediction of the CCJ model continued to increase as the shear rate decreased. Moreover, at the high shear rate area, the prediction of the CCJ equation deviated from the experimental data, while the new equation prediction was considered to match the experimental data better.

### 3.3. Yield Stress

Yield stress, as a key parameter of rheological properties in suspensions, can provide information about the performance of the ER fluids depending on electrical field strength, volume fraction, and dielectric properties of the particles. Many test methodologies have been applied to study yield stress characteristics, corresponding to different yield stress values including dynamic, elastic, and static yield stresses [118].

**Figure 12:**
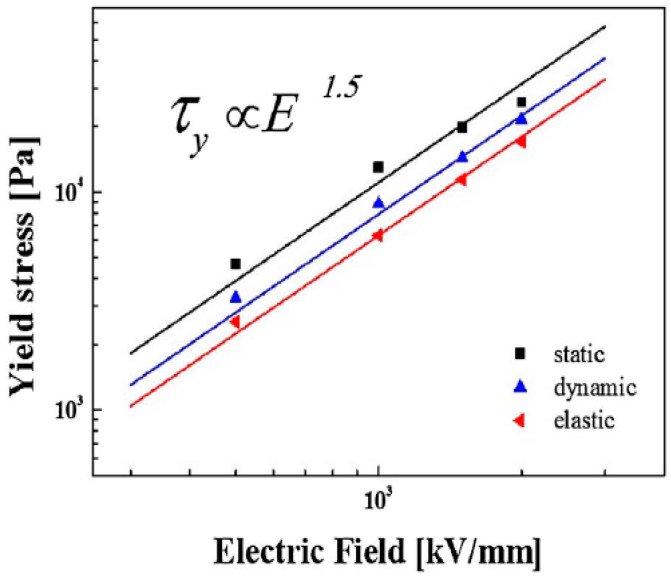
Static, dynamic, and elastic yield stresses as a function of electrical field strength for PANI-nanotube-based ER suspension, reprinted with permission from [119]. Copyright Elsevier, 2017.

As shown in Figure 12, Lim et al. [119] studied dynamic, static, and elastic yield stresses vs. electrical field strength for a PANI-nanotube-based ER suspension, estimated from controlled shear rate, controlled shear stress, and dynamic oscillatory frequency tests, respectively. The results showed that the static yield stress had the highest value because the static state that included more aggregate without shear rate control, as compared to the ER fluid under the dynamic condition. The elastic yield stress is obtained from the maximum shear stress required to achieve full recovery after the shear stress is removed, which is the lowest. Nonetheless, these three types of yield stress as a function of electric field strength presented equivalent behaviors.

**Figure 13:**
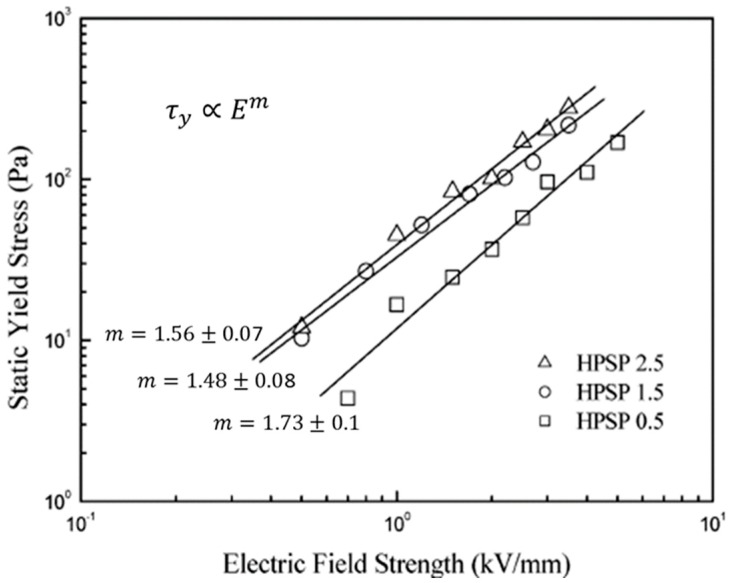
Yield stress vs. electrical field strength for highly substituted potato starch phosphate (HPSP)-based ER suspension with different molar ratios, reprinted with permission from [124]. Copyright American Chemical Society, 2005.

As mentioned above, the electric field strength dependent yield stress has been the focus of many studies on ER characteristics. Klingenberg et al. [6] introduced an electrostatic polarization model and calculated the functional relation between yield stress and electrical field strength as follows:

(6) $$\tau_{0}=18\varphi \epsilon_{0}\epsilon_{e}\beta^{2}E_{0}^{2}f_{m}\left[1-\frac{{\left(\pi /6\right)}^{1/2}}{\left(L/a\right)\tan\theta_{m}\emptyset^{1/2}}\right]$$

where φ represents a volume fraction and *L* represents the electrode separation. $f_{m}$ and $\theta_{m}$ are the maximum dimensionless restoring force and angle, respectively. This expression means that the dynamic yield stress is essentially proportional to the squared electrical field strength. However, although some experiments confirmed this result [120,121], the effects of the particular material, concentration, shape, and electric field strength will cause the index to deviate from 2.0 [122,123]. As shown in Figure 13, Sung et al. [124] observed that the yield stress of a highly substituted potato starch phosphate (HPSP)-based ER fluid was not proportional to *E*^2^, which deviated from the polarization model because of the non-spherical shape of HPSP.

Concurrently for conducting suspensions under a high electric field strength, the ER characteristic would be affected by a conductivity mismatch and an interaction between dispersed particles and dispersing oil, rather than by their dielectric constant mismatch. Davis et al. [125] investigated the effect of field-dependent fluid conductivity and proposed that the yield stress appeared to approach $E^{3/2}$ under high electric field strength (consistent with Figure 12) [126].

**Figure 14:**
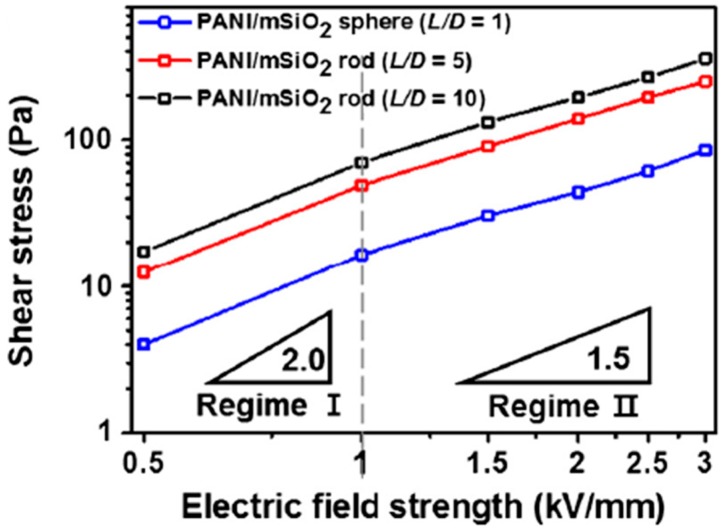
Yield stress of PANI/mSiO_2_-based ER suspension as a function of electrical field strength, reprinted with permission from [127]. Copyright Elsevier, 2016.

Moreover, as shown in Figure 14, the yield stresses of PANI/mSiO_2_-based ER suspensions can be separated into two different areas in the entire electric field strength ranges [127]. Choi et al. [128] proposed a critical electrical field strength (*E_c_*) and developed the following simplified equation to represent a hybrid yield stress for a wide electrical field strength area:

(7) $$\tau_{y}=\alpha E^{2}\left(\frac{\tanh\sqrt{E/E_{c}}}{\sqrt{E/E_{c}}}\right)$$

*E_c_* could be obtained from an intersection between two power-law slopes for a low and a high electrical field area. The parameter *α* is dependent on a particle volume concentration and a dielectric constant of the ER suspension. Equation (7) contains two limiting behaviors of yield stress: $E^{2}$ for $E\ll E_{c}$ and $E^{1.5}$ for $E\gg E_{c}$. As presented in Figure 14, the ER characteristics of PANI/mSiO_2_ corresponded to the non-linear conductivity model where the indices are 2 below *E_c_* and 1.5 above *E_c_*. Many other studies have proved the rationality of this equation [129,130].

## 4. Dielectric Analysis

**Figure 15:**
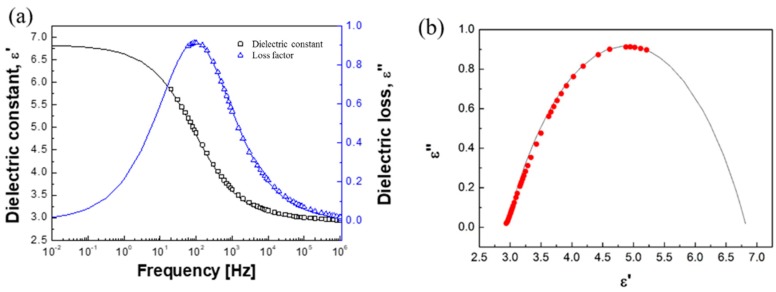
(**a**) Dielectric spectrum and (**b**) Cole–Cole plot of silica/POA based ER suspension, reprinted with permission from [82]. Copyright IOP Publishing, 2017.

A high dielectric constant shows a useful effect on the ER response because of the more effectively achievable polarizability. In general, the dielectric property is measured by an inductance capacitance resistance (LCR) tester. Figure 15 represents both the dielectric spectrum and the Cole–Cole curve of POA-silica composites [82]. The permittivity is frequency-dependent and can be expressed in its complex form involving permittivity ($\varepsilon^{\prime }$) and the loss factor ($\varepsilon^{\prime \prime }$): $\varepsilon^{*}=\varepsilon^{\prime }+\varepsilon^{\prime \prime }$. The polarizability is described as the permittivity difference of $\Delta \varepsilon =\varepsilon_{0}-\varepsilon_{\infty }$ and relaxation time ($\lambda =\frac{1}{2}\pi f_{max}$), which are obtained by fitting the dielectric spectra with the Cole–Cole formula.

(8) $$\varepsilon^{*}=\varepsilon^{\prime }+i\varepsilon^{\prime \prime }=\varepsilon_{\infty }+\frac{\Delta \varepsilon }{{\left(1+i\omega \lambda \right)}^{1-\alpha }}$$

In Equation (8), *ω* is a frequency and (1 − *α*) expresses the relaxation time distribution for a whole frequency area. It has been observed that a large ER efficiency is related to a large ∆*ε* and a dielectric loss peak is placed between 10^2^ and 10^5^ Hz. When *α* is zero, Equation (8) is reduced to the Debye’s single relaxation time model. In Dong ’s report [103], Equation (8) was used to compare different sizes of poly[*p*-vinylbenzyl trimethylammonium] based PILs possessing a similar diameter but different counter-anions. The results are shown in Table 1, with the counteranions of TfO, ∆*ε* having the largest value. Furthermore, in the subsequent ER response experiment, the PILs with TfO also exhibited the best ER effect, thereby showing that PILs with a smaller volume or size of counter-ions demonstrated a higher ER response.

## 5. Engineering Applications

With yield stress generated under external electrical field strengths applied, ER fluids have the advantages of reversible continuous control and fast response and so have been widely adopted in vibration control, tactile display, sensor, and other applications for different modes. In a flow mode, two electrodes are set, and the vibrational control is realized by tuning the fluid motion between the two electrodes. In a shear mode, one electrode rotates freely relative to the other, thereby controlling the vibration by tuning the shear force. In a squeeze mode, the electrode gap becomes larger and the ER suspension is extruded by the normal force.

Further detailed applications using ER fluids include the torsional vibration clutch, brake, damper, optical material polishing, tactile devices, and so on. The controllable ER characteristics can be used as power transfer agents with functions similar to friction in conventional clutches; thus, much research has been devoted to the application of this special object in automotive clutches. Madeja et al. [131] investigated the practical ranges of a hydrodynamic clutch by applying an electric field,. To control braking force distribution, Choi et al. [132] introduced a vehicle ER valve-based anti-block brake system (ABS). Until now, ER fluids have relatively low yield stress under 8 kPa; thus, they are not used as hydraulic actuators. However, with the development of giant ER fluids [133], because the yield stress of the ER actuator can reach 130 kPa, it is expected to produce ER actuator.

An ER fluid can be precisely controlled by tuning the electrical field intensity; thus, it can also be used in polishing the tactile devices of optical materials. Kuriyagawa et al. [16] used an ER solution for the first time in the fabrication of optical lenses. Compared with MR polishing, ER polishing has the advantages of a uniform electric field and simple electrode structure. Su et al. [134] used an ER fluid to fabricate wheeled integrated electrodes for micro-optical components.

Moreover, Taylor et al. [135] developed a force display device using a 1-degree of freedom (1-DOF) ER brake. Recently, Han et al. [136] reported a 4-DOF haptic device using ER fluids for a minimally invasive surgery.

## 6. Conclusions

In this study, we discussed various modified and unmodified conductive polymers, their composites with special structural properties (fiber, core-shell spherical or non-spherical particles, and so on), and their ER responses. Considerable progress has been made in the introduction of new and synthetic methods, and in their corresponding ER characteristics. ER materials with ideal chemical and physical characteristics can be prepared by interfacial oxidation, micro-emulsion, Pickering emulsion, polymer grafting, the co-precipitation hydrothermal method, the sol-gel method, and other methods. Among these, the study of PANI and PPy is the most extensive and comprehensive, not only in terms of the morphology and properties of the polymers themselves, but also in terms of the results of their combination with other organic or inorganic compounds. In recent years, many new conductive polymers, such as PDA, PDPA, PIN, and POA, and their composites with different conductivity properties, have been widely used in ER fluids.

The power law between yield stresses and electric field strengths of various conducting polymer- based ER fluids, with its exponent between 1.5 and 2.0, is explained based on both a polarization model with an exponent of 2.0 and a conduction model with an exponent of 1.5. Nonetheless, to cover various conducting polymer-based ER suspensions in which the exponents do not follow one of the two mechanisms mentioned above, a universal yield stress equation was designed to properly correlate their dependence. In addition, both the CCJ and four-parameter models exhibited better fitting performance for the shear stress curve of the ER suspensions under applied electrical fields in the range of shear rate as compared with that of the traditional Bingham fluid model. Finally, recent applications of ER fluids, such as in damper systems, control systems, haptic devices, polishing systems, and so on, were summarized for potential commercialization of various engineering devices.

## Figures and Tables

**Table 1:** Dielectric properties of PIL suspensions [103].

Sample	$\varepsilon_{0}$	Δε	λ
P[VBTMA][PF_6_]	4.70	1.70	0.002
P[VBTMA][BF_4_]	5.01	1.88	0.0061
P[VBTMA][TFSI]	5.14	1.96	0.0009
P[VBTMA][TfO]	5.11	2.01	0.00049
